# Unveiling the Morphostructural Plasticity of Zoonotic Sporotrichosis Fungal Strains: Possible Implications for *Sporothrix brasiliensis* Virulence and Pathogenicity

**DOI:** 10.3390/jof9070701

**Published:** 2023-06-25

**Authors:** Dario Corrêa-Junior, Iara Bastos de Andrade, Vinicius Alves, Igor Avellar-Moura, Vanessa Brito de Souza Rabello, Alessandro Fernandes Valdez, Leonardo Nimrichter, Rosely Maria Zancopé-Oliveira, Glauber Ribeiro de Sousa Araújo, Rodrigo Almeida-Paes, Susana Frases

**Affiliations:** 1 Laboratório de Biofísica de Fungos, Instituto de Biofísica Carlos Chagas Filho, Universidade Federal do Rio de Janeiro, Rio de Janeiro 21941-853, Brazil; dariojunior@biof.ufrj.br (D.C.-J.); iara.bastos@biof.ufrj.br (I.B.d.A.); viniciusalves@biof.ufrj.br (V.A.);; 2 Laboratório de Micologia, Instituto Nacional de Infectologia Evandro Chagas, Fundação Oswaldo Cruz, Rio de Janeiro 21040-900, Brazil; vanessabritorabello@gmail.com (V.B.d.S.R.); rodrigo.paes@ini.fiocruz.br (R.A.-P.); 3 Instituto de Microbiologia Paulo de Góes, Universidade Federal do Rio de Janeiro, Rio de Janeiro 21040-900, Brazil; 4 Rede Micologia RJ, FAPERJ, Rio de Janeiro, Brazil

**Keywords:** biophysics, one health, virulence factors, biofilm, chitin, lipid bodies

## Abstract

Sporotrichosis is a fungal infection caused by *Sporothrix* species, with *Sporothrix brasiliensis* as a prevalent pathogen in Latin America. Despite its clinical importance, the virulence factors of *S. brasiliensis* and their impact on the pathogenesis of sporotrichosis are still poorly understood. This study evaluated the morphostructural plasticity of *S. brasiliensis*, a fungus that causes sporotrichosis. Three cell surface characteristics, namely cell surface hydrophobicity, Zeta potential, and conductance, were assessed. Biofilm formation was also analyzed, with measurements taken for biomass, extracellular matrix, and metabolic activity. In addition, other potential and poorly studied characteristics correlated with virulence such as lipid bodies, chitin, and cell size were evaluated. The results revealed that the major phenotsypic features associated with fungal virulence in the studied *S. brasiliensis* strains were chitin, lipid bodies, and conductance. The dendrogram clustered the strains based on their overall similarity in the production of these factors. Correlation analyses showed that hydrophobicity was strongly linked to the production of biomass and extracellular matrix, while there was a weaker association between Zeta potential and size, and lipid bodies and chitin. This study provides valuable insights into the virulence factors of *S. brasiliensis* and their potential role in the pathogenesis of sporotrichosis.

## 1. Introduction

Sporotrichosis is a subcutaneous infection caused by dimorphic fungi belonging to the genus, *Sporothrix* [1]. *Sporothrix brasiliensis*, the most virulent species within the genus, has been identified as the major agent responsible for the zoonotic transmission of sporotrichosis in Brazil [2]. For the establishment of sporotrichosis, a dimorphic transition is necessary, where the *Sporothrix* conidia transform into yeast-like cells [3]. The host immune system recognizes various fungal components including mannose, rhamnomannans, and β-glucans and, if phagocytosis fails to occur, the microorganism can more easily establish itself within the host, resulting in an intensified infectious process [4]. Phagocytosis assays showed that macrophages were able to internalize both conidia and yeasts of *S. schenckii*. Recognition of conidia involved the participation of mannose receptors and resulted in the development of a Th1 response. Conversely, yeast recognition involved the participation of complement receptors [5].

Considering that *S. brasiliensis* can be transmitted from animals to humans, surveillance efforts must adopt the One Health perspective, including the environment, in addition to people and animals. This is crucial in urban areas with high population density, to prevent and control this major zoonosis in endemic regions [1]. In fact, sporotrichosis is one of the most common skin infections in infectious dermatology in Rio de Janeiro, Brazil, and it is intrinsically related to animal and environmental factors. Although the incidence of this mycosis is increasing, knowledge about the eco-epidemiology of their agents remains limited [5,6]. According to the Brazilian Institute of Geography and Statistics (IBGE), Brazil has the second largest pet population in the world, with 22.1 million cats and 52.2 million dogs [7]. Hence, studies that evaluate the mechanisms by which the fungus causes disease in these hosts are of paramount importance.

Sporotrichosis clinical presentations may vary according to the patient’s immune status, fungal pathogenicity, thermal tolerance of the strain, among other factors [8]. Among the fungal-related factors, which include inoculum size and virulence, some studies have shown differences between the main human pathogenic species of the genus *Sporothrix*: *S. brasiliensis* is widely acknowledged as the most virulent, while *S. globosa* is the least virulent. *S. schenckii*, on the other hand, has an intermediate virulence phenotype [8,9]. One of the most studied *Sporothrix* virulence factors is melanin production. Pathogenic *Sporothrix* species can produce melanin that guarantees protection against antifungal drugs and phagocytosis [10]. Virulence aspects must be studied continuously because the increase in fungal virulence poses threats to human health [11].

The cell wall (CW) is one of the main components of the fungal cell structure, with several biological functions related to morphology, integrity, pathogenicity, and virulence [12]. It is a complex, dynamic, and multilayered structure, located outside the plasma membrane, which participates in the initial interaction between the fungus and the environment. It is also a permeable barrier, with functions related to nutrition and protection of the protoplasm against physical or osmotic injuries [13]. In addition to chitin and β-glucans, the fungal cell walls can also contain proteins and other carbohydrates [14]. A comparison of the chemical composition and cell wall structure of *S. schenckii* and *S. brasiliensis* revealed that both fungi exhibit a bilayer wall with the outermost layer containing a peptidorhamnomannan fibrillar component, while chitin, β-1,3, β, 1,6-glucans, and glycogen particles are present in the innermost layer close to the plasma membrane [15]. In addition, *S. brasiliensis* contains more chitin and rhamnomannan polymers [16]. The CW composition of three strains of *S. schenckii* and two strains of *S. brasiliensis* exhibiting different levels of virulence in a murine model of infection were compared, confirming previous results for the species [15]. More recently, it was reported that culture media influence changes in cell wall composition and structure, as well as virulence in *S. schenckii* and *S. brasiliensis*, but not in *S. globosa* [17]. 

*S. brasiliensis* is known to produce trehalose, a disaccharide involved in resistance to osmotic stress, resulting in cell wall remodeling. This factor may be related to *Sporothrix* environmental adaptation, reflecting in its virulence [18]. In addition, *S. brasiliensis* and *S. schenckii* have qualitative similarities between polysaccharide and amino acid CW contents, with some quantitative differences depending on the culture incubation time [16]. The composition changes depending on cell morphology, with the percentage of *N*-acetylglucosamine, the basic unit of chitin, being slightly higher in similar cells the yeast *S. schenckii*, when compared to the same morphotype of *S. brasiliensis* [19,20].

Another important fungal virulence factor is biofilm formation, which allows microorganisms to survive in hostile conditions [21,22]. Biofilms are dynamic communities that can protect microorganisms from host defenses and increase drug tolerance. In addition, they are characterized by an extracellular matrix that can impede the penetration and diffusion of antimicrobial substances [23]. *Sporothrix* species are known to form biofilms [24], which may contribute to their pathogenicity [25]. While *S. brasiliensis* and *S. globosa* produce characteristic biofilms, those formed by *S. schenckii* appear to have less matrix material, which is primarily composed of carbohydrates and proteins, similar to *Candida* and *Aspergillus* biofilms [25]. 

Despite the existing knowledge in this area, there is a lack of studies that focus on comparing the morphostructural aspects of the sporotrichosis agents, particularly those isolated from diverse hosts. Therefore, the aim of this study was to evaluate morphostructural aspects of *S. brasiliensis* strains from domestic outbreaks of cat-transmitted sporotrichosis in Rio de Janeiro, Brazil. The study aimed to identify potential host associations and to explore the relationships among various cellular characteristics, with insights into the fungal virulence.

## 2. Materials and Methods

### 2.1. Fungal Strains

Twenty-four *S. brasiliensis* strains from human patients and their respective cats with sporotrichosis treated at the Instituto Nacional de Infectologia Evandro Chagas (INI-Fiocruz), previously identified by a species-specific PCR [26] were used. These well-characterized strains are deposited at the Fungal Collection “Coleção de Fungos Patogênicos”, which is recognized by the World Data Centre for Microorganisms (WDCM 951). All humans reported a history of contact with the sick cat before the onset of symptoms, as reported [27]. All strains were cultured for seven days on brain–heart infusion (BHI) agar (Difco, Franklin Lakes, NJ, USA) at 35 °C to obtain yeast cells and on potato dextrose agar (PDA—Difco, Franklin Lakes, NJ, USA) at 25 °C to obtain conidia, then washed again in phosphate-buffered saline—pH 7.2 (25 °C) (PBS) and the inoculum proposed in each of the following experiments was performed.

### 2.2. Evaluation of Cell Surface Hydrophobicity 

Cell surface hydrophobicity (CSH) was measured by a two-phase water–octane assay. A cell suspension (1.2 mL) containing 10^8^ yeasts/mL was washed with PBS and stirred vigorously with 0.3 mL of octane (Sigma-Aldrich, St Louis, MO, USA). The two solvents were allowed to separate for 15 min at room temperature. The absorbance at 600 nm (ABS_600_) of fungal cells in PBS with no octane overlap was used as a control. The percentage of fungal cell exclusion from the aqueous phase (% change in ABS_600_) corresponding to the relative cellular hydrophobicity was calculated as follows: ([ABS_600_ from control—ABS_600_ after octane overlay]/ABS_600_ from control) × 100. As previously reported, high, moderate, and low CSH correspond to respective changes of 80–100%, 20–80%, and 0–20% in ABS_600_, respectively [28,29].

### 2.3. Evaluation of Cellular Electronegativity 

The Zeta potential (ζ), particle mobility and shift frequency of samples were calculated in a Zeta potential analyzer (NanoBrook Omni Zeta Potential Analytical Instruments Particle Sizer, Brookhaven Instruments Corp., Holtsville, NY, USA). This is a measurement of charge (in millivolts) defined as the potential gradient that develops across the interface between a boundary liquid in contact with a solid and the mobile diffuse layer in the body of the liquid. The Zeta potential was evaluated with a yeast cells suspension (1.5 mL containing 10^6^ yeasts) in a solution of 1 mM NaCl in pure water at 25 °C, pH 6.0. All measurements were performed in 10 cycles. In addition to the Zeta potential values, conductance was evaluated, that is, the application of electrical force, which is directly proportional to the number of ions presented by these samples [30,31].

### 2.4. Biofilm Formation on Polystyrene

Biofilm formation on a polystyrene substratum was performed as described previously for *Aspergillus fumigatus* [32]. The experiment was performed on flat-bottom 96-well polystyrene microtiter plates (Kasvi, São José dos Pinhais, PR, Brazil) where 100 µL of BHI broth (Sigma-Aldrich, St Louis, MO, USA) containing approximately 1 × 10^6^ cells/mL was added to each well. Plates were incubated for 7 days at 35 °C, without agitation. Negative controls consisted of medium alone and were set up in parallel. Subsequently, wells were washed three times with 200 µL of PBS to remove nonadherent cells [29,32]. The tests below were carried out, in biological and experimental triplicate, to analyze the biomass, extracellular matrix and viability of the biofilms.

#### 2.4.1. Biomass Quantification 

Biofilms were fixed with 200 µL of 99% methanol (Sigma Aldrich, St Louis, MO, USA) for 15 min. Then, the supernatants were discarded, microplates were air-dried for 5 min and 200 µL of a 0.5% crystal violet solution (stock solution diluted in PBS; Sigma Aldrich, St Louis, MO, USA) were added to each well with a subsequent incubation for 20 min at 25 °C. Dye was removed by washing the material with distilled water and the biomass was decolorized with 200 µL of 33% acetic acid (Sigma Aldrich, St Louis, MO, USA) (*v*/*v*) for 5 min. Aliquots of 100 µL of the acetic acid solution were transferred to a 96-well plate (Jet Biofil, Huangpu, Guangzhou, China) and the absorbance was measured at 590 nm using a microplate reader (SpectraMax M3; Molecular Devices, SanJosé, CA, USA). Classification regarding biomass production was carried out as follows: absorbance values lower than 0.44 correspond to low biomass production, values from 0.44 to 1.17 correspond to moderate biomass production, and values higher than 1.17 correspond to high biomass production [33]. 

#### 2.4.2. Cellular Viability

Mitochondrial activity was determined with the CyQUANT™ XTT assay (Thermo Fisher, Waltham, MA, USA). A mixture of XTT reagent and electron coupling reagent was prepared at a ratio of 6 to 1. Seventy microliters of the mixture were added to each well. Then, the plate was incubated at 37 °C for 4 h in the dark in a 5% CO_2_ incubator. The optical densities (OD) were determined using the SpectraMax^®^ Plus 384 Microplate Reader (SpectraMax M3; Molecular Devices, SanJosé, CA, USA) at 450 nm and 660 nm [31,34]. Mitochondrial activity was expressed by the decrease in OD (450–660 nm), discounting the average of the ODs of the controls [35]. The isolates were categorized into three groups based on their metabolic activity: low metabolic activity, moderate metabolic activity, and high metabolic activity. The classification was determined using XTT with respective cut-offs of <0.097, 0.097–0.2, and >0.2 [33].

#### 2.4.3. Characterization of Extracellular Matrix Production

Cells were stained with 200 µL of 0.1% safranin (dissolved in PBS; Sigma-Aldrich, St Louis, MO, USA) for 5 min at room temperature. Then, wells were washed with PBS to remove excess stain until supernatants became clear. The extracellular matrix was discolored with 200 µL of 30% (*v*/*v*) acetic acid for 5 min. Aliquots of 100 µL of the supernatants were transferred to a new 96-well plate (Jet Biofil, Huangpu, Guangzhou, China) and the absorbance was measured at 530 nm using the SpectraMax M3 microplate reader, as described previously [31,36].

### 2.5. Detection of Lipid Bodies in the Fungal Cell 

Eukaryotic cells are able to produce lipid bodies, made up of neutral lipids, which have a role in a variety of cellular functions such as ergosterol biosynthesis. In this context, fungi (10^6^ yeasts/mL) were fixed in paraformaldehyde (Sigma Aldrich, St Louis, MO, USA), stained with Nile red (Sigma Aldrich, St Louis, MO, USA) at 5 µg/mL for 30 min at room temperature. Yeasts were treated with PBS alone as an autofluorescence control. Subsequently, the fungi were washed three times in PBS and analyzed in a flow cytometer (BD Biosciences, San Jose, CA, USA). The mapped population (*n* = 10,000 events) was analyzed for size and log red fluorescence using a single parameter histogram. Results were expressed as mean of fluorescence intensity (MFI), as described [29].

### 2.6. Quantification of Chitin in the Cell Wall 

Yeast suspensions were prepared as described above and treated with Uvitex 2B (Polysciences Inc., Warrington, PA, USA) to detect chitin in their cell walls. In brief, 1 µL of 10 µg/mL Uvitex2B was added to 1 mL of the conidial suspension. This mixture was incubated at 35 °C for 60 min. Then, they were washed three times in PBS, analyzed in a flow cytometer (BD Biosciences, San Jose, CA, USA), and the results were expressed as described previously [29,37]. 

### 2.7. Ultrastructural Analysis of the Cell Surface 

Scanning electron microscopy (SEM) was conducted by washing the yeasts three times in PBS and subsequently fixing them in 2.5% glutaraldehyde type 1, diluted in 0.1 M sodium cacodylate buffer, for 1 h at room temperature. Then, the structures were washed in 0.1 M sodium cacodylate buffer containing 0.2 M sucrose and 2 mM MgCl_2_, adhering to cover slips coated with 0.01% poly-L-lysine (Sigma Aldrich, St Louis, MO, USA) for 20 min. With the structures adhered, they were dehydrated in a series of freshly produced solutions of graded ethanol (30, 50 and 70%, for 5 min/step, then 95% and twice 100%, for 10 min/step). The samples were then subjected to critical point drying (EM CPD 300, NY, Leica) immediately after dehydration, metal stumps were mounted, coated with a 15–20 nm gold layer (Balzers Union FL-9496, Balzers, Liechtenstein), and viewed under an SEM microscope (FEI Quanta 250, Hillsboro, OR, USA), operating at 10–20 kV. The SEM images were analyzed in the ImageJ software bundled with Java 1.8.0_172 [38], where they were converted into 8-bit, then the function Threshold was utilized. Subsequently, the cells were segmented and measured, and a table with the area values of each cell was produced. The SEM scale bar was measured, and its value divided by the number of pixels, allowing the determination of the area value in square micrometers [31].

### 2.8. Statistical Treatment

All experiments were performed in triplicate, in three independent experimental sets, except when strictly described before, and the results were expressed as mean or mean ± standard deviation, whenever applicable. The results were analyzed using the GraphPad Prism 9.5 software (La Jolla, San Diego, CA, USA), with *p*-values less than 0.05 to determine the significance. Non-parametric tests were used as appropriate and necessary in the different analyzes and comparisons among the samples seized. The overall analysis of all virulence-related phenotypes was performed through a heat map constructed with the Heatmapper tool [39]. The correlation between the expression of different phenotypes by the same strain was assessed using the Spearman’s correlation, in the GraphPad Prism 9.5 software. 

## 3. Results

### 3.1. Cell Surface Properties 

**Figure 1:**
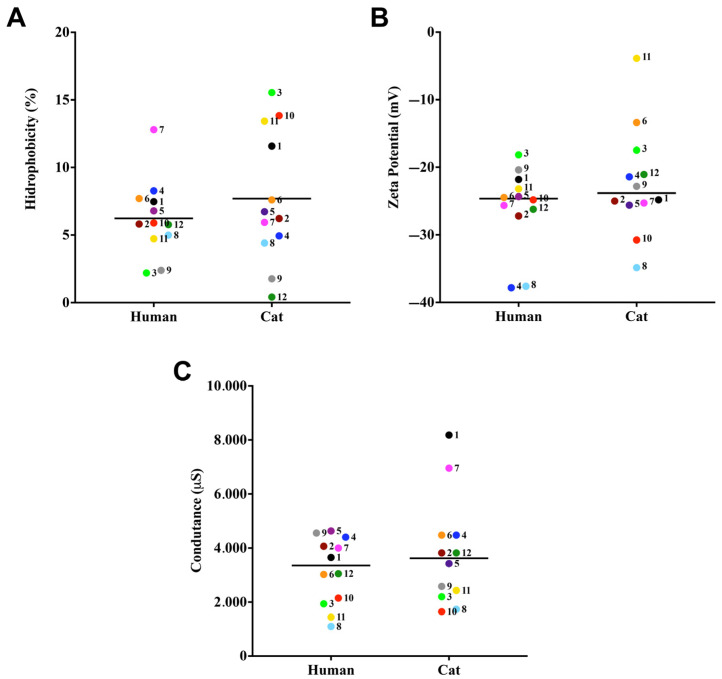
Cell surface properties of cat- and human-derived cases of *S. brasiliensis*. The CSH was evaluated by the two-phase partition (water-octane) method. Zeta potential and conductance was evaluated using a Zeta potential analyzer. CSH (**A**), Zeta potential (**B**), and conductance (**C**). Each dot represents the mean value of three independent experiments performed in technical triplicates. Dot colors and numbers after dots represent the different familial outbreaks. Black lines represent the median value of each group of strains.

In this set of experiments, three cell surface characteristics of the clinical isolates comprising the *S. brasiliensis* were evaluated: CSH, Zeta potential (ζ), and conductance. The partition of fungal cells in a water–octane solution revealed a strain-specific pattern of CSH in our collection sample. In this context, among the 24 clinical isolates studied, all exhibited low CSH (Figure 1A). In addition, the median CSH values of grouped strains according to their host of isolation did not present significant differences (Figure 1A). Regarding the ζ, all isolates exhibited electronegative charge, with values ranging from −37.8 to −3.8 mV (Figure 1B), with no statistically significant differences observed among cat- and human-derived isolates (*p*-value >0.05, Tukey’s multiple comparisons test). The conductance of *S. brasiliensis* ranged from 1094 to 8182 µS (Figure 1C). Only case 4 did not show a significant difference between the analyzed strains, all the other 11 cases had <0.0001 difference between the strains (Tukey’s multiple comparisons test).

### 3.2. Biofilm Properties 

**Figure 2:**
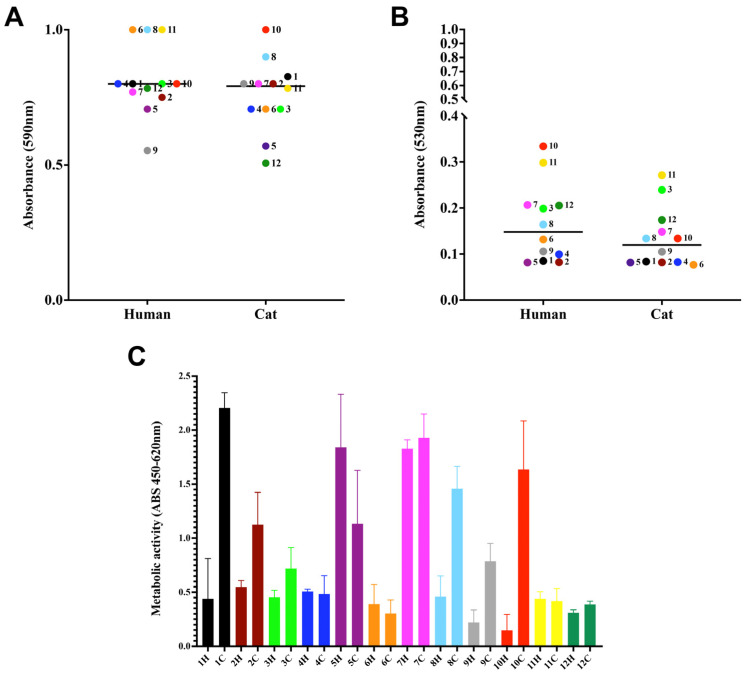
Biofilm formation by *S. brasiliensis* on a polystyrene surface. Yeasts (10^6^ cells in 100 µL) were placed to interact with polystyrene for 7 days at 35 °C. Fungal biomass via incorporation of crystal violet at 590 nm (**A**), extracellular matrix via staining the non-fixed biofilms with safranin at 530 nm (**B**), and viability (**C**) via the reduction of assay. The results are expressed as median (**A**,**B**) and SD (**C**).

In this set of experiments, three distinct biofilm parameters were analyzed: biomass via the incorporation of crystal violet dye (Figure 2A), extracellular matrix via the absorption of safranin (Figure 2B), and metabolic activity via the reduction of XTT (Figure 2C). All samples produced moderate biomass and did not show differences among human and cat-derived cases. Regarding the production of extracellular matrix, only case 10 was significantly different among human and cat strains (*p*-value < 0.0001). The viability of cells forming the biofilm did not show a significant difference, except for cases 1, 8, and 10 (*p*-value: <0.0001, 0.0006, and <0.0001, respectively).

### 3.3. Lipid Body and Chitin Contents

**Figure 3:**
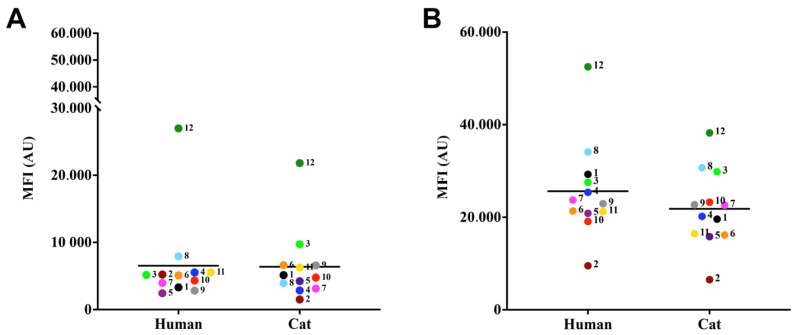
Relative detection of lipid bodies (**A**) and chitin (**B**) produced by *S. brasiliensis*. For lipid bodies detection, fungal cells were labeled with the fluorochromes, Nile red (**A**) and Uvitex 2B, and evaluated via flow cytometry. The results were expressed as median of fluorescence intensity (MFI) of *S. brasiliensis* fluorescent cells of three independent experiments.

The lipid body results showed that these structures were detected in a similar way regarding MFI (means of 26,981.3 ± 1501.6) (Figure 3A). No difference was observed between the lipid body measurements of the *S. brasiliensis* isolates studied (*p*-value > 0.05; Tukey’s multiple comparisons test). Regarding chitin content, all isolates presented high percentages of calcofluor-labeled cells (MFI overall mean of 6511.3 ± 52,509.6) (Figure 3B), and no statistically significant differences were found among the cases (*p*-value > 0.05; Tukey’s multiple comparisons test).

### 3.4. Ultrastructural Analysis of the Cell Surface

**Figure 4:**
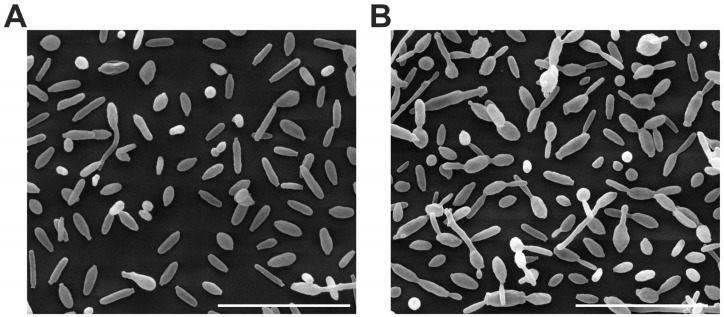
Scanning electron microscopy (SEM) image of *S. brasiliensis* yeasts; 6H (**A**) and 6C (**B**). Scale bar: 20 µm.

All yeast from paired cases observed was similar of size and morphology. Figure 4 shows an SEM image of one of all the analyzed cases of *S. brasiliensis.*

**Figure 5:**
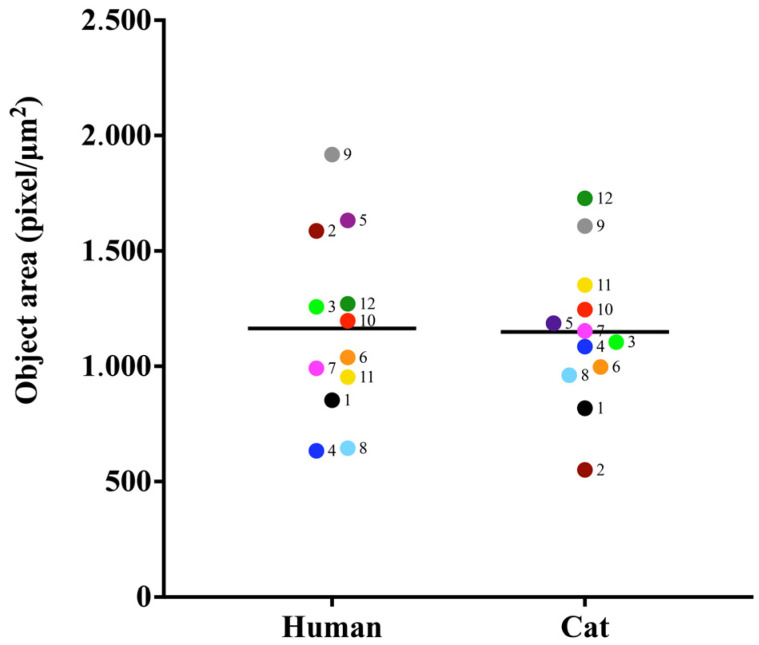
Object area analysis in (pixel/µm^2^) of *S. brasiliensis* yeasts. Results are expressed as the median of the measurements.

The strains studied were of varying sizes grown under the same conditions. Samples from case 2 showed a significant difference between them (*p*-value <0.0001) (Figure 5).

### 3.5. Comparative Analysis of Morphostructural Properties 

**Figure 6:**
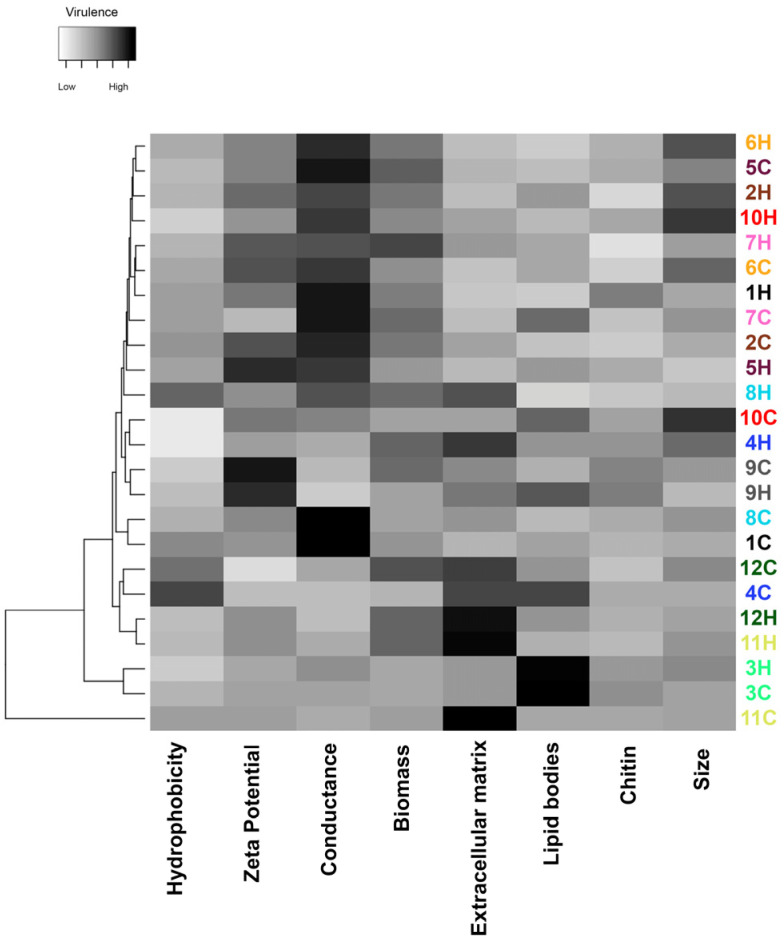
Global expression of putative virulence-associated factors of *S. brasiliensis* strains from 12 familial outbreaks of sporotrichosis. The gray scale in the heatmap ranges from low (white) to high virulence (black). Different strains are represented in the lines of the heatmap, and the different virulence factors herein studied in the columns of the heat map. Strains were grouped in a dendrogram reflecting the similarity between the virulence-related phenotypes of each strain. Colors of the strain identification numbers represent the different familial outbreaks.

**Figure 7:**
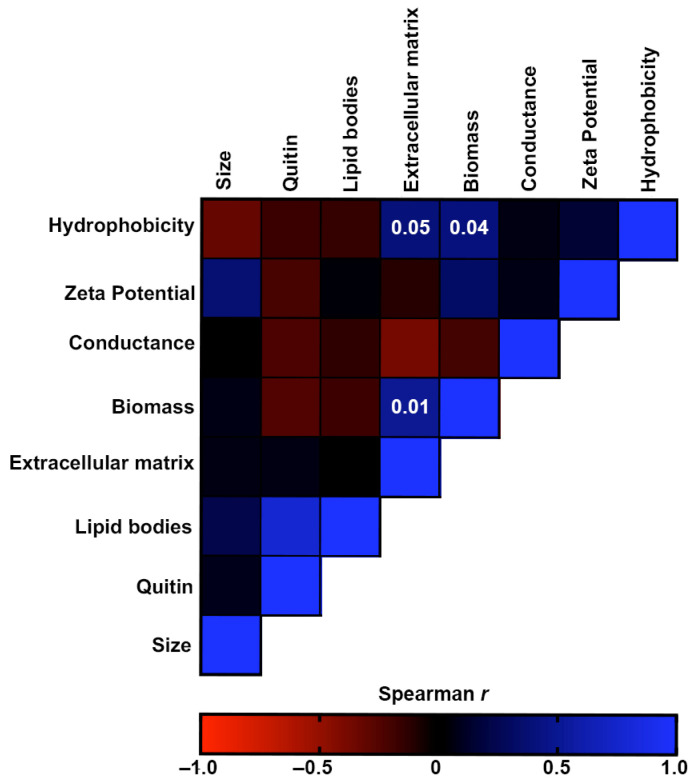
Correlation analyses of the virulence-associated factors produced by the *S. brasiliensis* strains from 12 familial outbreaks of sporotrichosis. The heat map represents the Spearman’s correlation coefficients of the associations. Red color represents negative monotonic correlations, whereas the blue color represents positive monotonic correlations. Probability values of statistically significant correlations (*p*-value < 0.05) are displayed within the respective squares of the heat map.

As depicted in Figure 6, the major phenotypic features potentially related to fungal virulence among the studied *S. brasiliensis* strains were Zeta potential, conductance, and extracellular matrix. The dendrogram clustered the strains according to their global similarity on the production of those studied factors (Figure 7). It is interesting to note that human- and cat-derived strains from outbreaks 3 and 9 clustered together, while strains from outbreaks 11 and 12 clustered very closely.

Figure 7 shows the correlation analyses of the morphostructural factors produced by the *S. brasiliensis* strains. CSH was positively associated with the production of biomass and extracellular matrix of biofilms. Moreover, these two biofilm characteristics were also positively associated. Although not significant, there was also a weak association between Zeta potential and cellular size (*p* = 0.37) and between lipid bodies and chitin (*p* = 0.77).

## 4. Discussion

*S. brasiliensis* is a good fungal model for One Health studies since it can be present in the environment as well as infect humans and animals [40]. Differences between human and animal isolates of *S. brasiliensis* can be attributed to specific host–pathogen interactions. Variations in immune responses between species can impact the behavior and characteristics of the fungus during infection [2,27]. Additionally, environmental factors in human and animal hosts can influence fungal behavior and gene expression, resulting in distinct morphostructural characteristics [41]. In addition, even if the isolate pairs derive from a single outbreak of sporotrichosis involving humans and cats, there is a possibility that the human became infected not by the cat, but from another source, environmental perhaps, and then they would present differences in their phenotypes. It is important to note that the primary objective of the study was not to investigate these differences, but rather to explore previously unstudied morphostructural features of *S. brasiliensis*. 

*Sporothrix brasiliensis* is the primary causative agent of zoonotic sporotrichosis in Brazil, and its incidence is also increasing in Latin America [42]. This species is of great importance as it causes a subcutaneous mycosis that affects both humans and animals [43]. Zoonotic sporotrichosis, at least in our country, is mainly caused by *S. brasiliensis* and not by other *Sporothrix* species [44]. Understanding the biology of *S. brasiliensis* is critical for enhancing the diagnosis, treatment, and prevention of sporotrichosis in humans [8]. It possesses morphostructural features that distinguish it from other *Sporothrix* species. Investigating these unique characteristics allows us to gain insights into the fungus–host interactions and the mechanisms of infection. There is a need to fill gaps in the scientific literature and expand our understanding of this species. It is essential to note that our study specifically focused on *S. brasiliensis* and its morphostructural characteristics [40].

In recent years, there has been an increase in the number of pets living with humans, raising the concerns about pathogens that can be zoonotically transmitted [40]. In Brazil, human sporotrichosis can be acquired from the environment or from bites or scratches of naturally infected cats. Moreover, these animals can transmit the disease to dogs or other cats [8]. In this study, we evaluated and compared *S. brasiliensis* strains from 12 familial outbreaks of cat-to-human-transmitted sporotrichosis. It is plausible that cats infected their owners in these outbreaks, although it is also possible that humans contracted sporotrichosis from an environmental source or from cats outside their households. The overall analysis of our findings suggests that, in at least five familial outbreaks, the morphophysiological characteristics of the strains obtained from both humans and cats residing in the same dwelling were highly similar, supporting the evidence of their zoonotic transmission. 

Species of the pathogenic clade of the *Sporothrix* genus have been used to investigate differentiation/dimorphism, pathogenesis, the enzymology and molecular mechanisms of wall glycoprotein assembly, the search of surface antigens, particularly adhesins, cell wall biogenesis and regulation, and most recently the cell compensatory responses to damage of the cell surface [45]. The *Sporothrix* species exhibit different levels of virulence, variable forms of transmission and geographic distribution [46]. The sporotrichosis pathogenesis depends on many essential steps, which begin with *Sporothrix* spp. interaction with host cells and molecules [47,48]. This event can be influenced by physicochemical properties of the fungal cell surface, such as hydrophobicity and electrical charge [47,48]. In fact, fungi and bacteria can modify their shape and surface structure as adaptive mechanisms for survival and dissemination in the environment or within the host [49]. The CSH is one of the main nonspecific factors involved in the adhesion of microbial cells to different biotic and abiotic substrates [50]. Our results showed, for the first time, differences in the CSH among clinical *S. brasiliensis* isolates studied, identifying the presence of low CSH among them. *Candida albicans* [51] and *Candida* non-*albicans* species present high CHS [29]. Zeta potential is a parameter that determines the electrophoretic mobility of a charged particle in a liquid medium and represents the electrical layer around the cell [52]. The fungal surface electronegativity is important for attraction and initial interaction in host cells [53]. All clinical isolates herein studied presented negative surface charge, exhibiting ζ values between −37.8 and −3.8 mV. This is the first time that the cell surface charge of *S. brasiliensis* is described. The cellular surface of fungal cells is generally negatively charged, as previously described for *C. albicans* and several other *Candida* species, *Cryptococcus neoformans*, *A. fumigatus*, and *A. niger* [29,53,54,55,56]. The negative charge observed in *S. brasiliensis* yeast cells may be attributed to the presence of melanin, a component of the fungal CW that has been shown to possess a negative charge [57]. The conductance importance in fungi is not well understood, but it is known that for other cell types, it interferes with the microenvironment [58]. 

Biofilms are highly dynamic cellular communities that play a crucial role in protecting microorganisms from host defenses and enhancing their resistance to drugs, probably via their extracellular matrix, which acts as a barrier that hinders penetration and diffusion of antimicrobial substances [25]. All samples produced biofilms with moderate biomass and high metabolic activity. The presence of an extracellular matrix, observed in all strains, provides stability, integrity, and strength to the biofilm architecture [25]. Hydrophobicity was directly and positively correlated with the production of biomass and extracellular matrix. This observation can be attributed to the role of CSH as one of the primary biophysical characteristics contributing to the adhesion of microbial cells to various biotic and abiotic surfaces [50]. For *A. fumigatus* biofilm, a correlation between metabolism, biomass, and hyphal development has previously been demonstrated [32]. 

The fungal CW is a dynamic structure, presenting continuous changes in composition and structural organization as the cell grows or undergoes morphological changes [13]. A comparison of the chemical composition and structure of *S. schenckii* and *S. brasiliensis* CW revealed that both fungi exhibit a bilayer wall with the outermost layer containing a fibrillar peptidorhamnomannan component, while chitin, β-1,3-, β-1,6-glucans, and glycogen particles are present in the innermost layer in the proximity of the plasma membrane [58]. Moreover, *S. brasiliensis* contains more chitin and rhamnomannan polymers [16]. The CW composition of three *S. schenckii* and two *S. brasiliensis* strains exhibiting different levels of virulence in a murine model of infection were compared, confirming previous results for the species [15]. All isolates presented high percentages of calcofluor-labeled cells in our study, but without differences between hosts. Neutral lipids, which can be stored inside lipid bodies in *Candida* cells, act as energy reservoirs and participate in membrane-formation and -maintenance [29]. For this reason, we investigated the presence of lipid bodies in *S. brasiliensis,* these structures were detected in a similar way regarding both analyzed parameters. Lipid bodies were directly correlated with chitin production. Possibly, the increase in cell size induced by the presence of lipid bodies allows for more surface area on the CW, thereby facilitating a greater deposition of chitin and other components.

Immune response mechanisms in infections are multiple due to the size and metabolic diversity of the parasites [49]. Fungi can induce morphological transitions that contribute to the evasion of the immune response, dissemination through the organism, and tissue invasion [58]. 

Fungal giant cells are specialized structures found in certain fungi, resulting from vegetative cell fusion or incomplete cell division. Their functions may include absorbing nutrients, defending against pathogens and predators, forming physical barriers, or releasing antimicrobial compounds [59,60]. *Cryptococcus neoformans* is an example of a fungus that forms giant cells, playing a role in host colonization and virulence [61,62]. The complete understanding of these cells is still the subject of research. *S. brasiliensis* yeasts vary in sizes when grown under the same conditions, demonstrating that *S. brasiliensis* isolates can adapt their cell size according to the physiological needs encountered. Zeta potential was directly correlated with the size, suggesting that cell size can change the contact surface of the cell with the medium in which it is inserted, changing the surface charge, a factor important for attraction and initial interaction [53].

In this study, we observed variations in morphostructural characteristics among *S. brasiliensis* isolates, suggesting the plasticity of these strains in establishing infections in humans and cats. Interestingly, these characteristics did not differ significantly between different hosts, aligning with the “one health” perspective observed in other pathogenic fungi [40]. While our main focus was not on establishing a direct correlation between virulence and morphostructural characteristics, our study fills a significant gap in the literature by exploring this previously unexplored aspect. This research represents the first application of these methodologies within the genus. Our findings highlight the importance of understanding the biology of *S. brasiliensis* for improving the diagnosis, treatment, and prevention of zoonotic sporotrichosis. The unique morphostructural features of *S. brasiliensis* provide valuable insights into its interactions with the host and the mechanisms of infection. Further research is needed to expand our knowledge of this species and its role in sporotrichosis. Overall, our study contributes to the broader understanding of *S. brasiliensis* as the primary causative agent of zoonotic sporotrichosis in Brazil.

## Data Availability

The data in this study are available in the presented manuscript.
